# Developmental Transcriptomics Reveals a Gene Network Driving Mimetic Color Variation in a Bumble Bee

**DOI:** 10.1093/gbe/evab080

**Published:** 2021-04-21

**Authors:** Sarthok Rasique Rahman, Tatiana Terranova, Li Tian, Heather M Hines

**Affiliations:** 1 Department of Biology, The Pennsylvania State University, University Park, Pennsylvania, USA; 2 Department of Biological Sciences, The University of Alabama, Tuscaloosa, Alabama, USA; 3 Department of Entomology, China Agricultural University, Beijing, China; 4 Department of Entomology, The Pennsylvania State University, University Park, Pennsylvania, USA

**Keywords:** bee, evolutionary genetics, pigmentation, melanin, transcriptomics, mimicry

## Abstract

A major goal of evolutionary genetics and evo-devo is to understand how changes in genotype manifest as changes in phenotype. Bumble bees display remarkable color pattern diversity while converging onto numerous regional Müllerian mimicry patterns, thus enabling exploration of the genetic mechanisms underlying convergent phenotypic evolution. In western North America, multiple bumble bee species converge onto local mimicry patterns through parallel shifts of midabdominal segments from red to black. It was previously demonstrated that a Hox gene, *Abd-B*, is the key regulator of the phenotypic switch in one of these species, *Bombus melanopygus*, however, the mechanism by which *Abd-B* regulates color differentiation remains unclear. Using tissue/stage-specific transcriptomic analysis followed by qRT–PCR validation, this study reveals a suite of genes potentially involved downstream of *Abd-B* during color pattern differentiation. The data support differential genes expression of not only the first switch gene *Abd-B*, but also an intermediate developmental gene *nubbin*, and a whole suite of downstream melanin and redox genes that together reinforce the observed eumelanin (black)-pheomelanin (red) ratios. These include potential genes involved in the production of insect pheomelanins, a pigment until recently not thought to occur in insects and thus lacking known regulatory enzymes. The results enhance understanding of pigmentation gene networks involved in bumble bee color pattern development and diversification, while providing insights into how upstream regulators such as Hox genes interact with downstream morphogenic players to facilitate this adaptive phenotypic radiation.

**Significance**

Bumble bees exhibit remarkable color pattern diversity that is primarily attributed to Müllerian mimicry. Previous research has identified the red-black mimetic color variation in bumble bee *Bombus melanopygus* to be driven by genetic changes in a major upstream developmental gene (*Hox* gene *Abd-B*) dictating segmental morphology. Using a stage-specific transcriptomic approach, we unravel how this gene ultimately drives the melanic pigmentation differences, identifying a suite of genes from the most upstream *Abd-B*, to intermediate developmental genes, and finally to a set of interacting downstream melanin and redox genes. These data expand our knowledge of evolutionary genetic processes underlying insect pigmentation and provide a toolkit of genes capable of driving parallel color variation across the bumble bee mimetic radiation.

## Introduction

Pigmentation traits have numerous adaptive roles in animals and as such, can achieve exceptional diversity in nature. Some of these selective pressures come from the myriad of physiological roles pigmentation plays, including innate immune defense (Whitten and Coates 2017), thermoregulation (Clusella Trullas et al. 2007), desiccation resistance, photo-protection (Hu et al. 2013), and parasite avoidance (Gillespie and et al. 1997). However, some of the most intriguing forces driving pigment diversity reflect its many roles in intraspecies and interspecies communication, from conspecific recognition and mate choice, to mimicry and crypsis. As such, genes involved in pigmentation are genomic targets of adaptation, and the frequent subject of evolutionary genetics research (Orteu and Jiggins 2020).

Genomic research on insect pigmentation has provided many insights into the types of genetic changes behind phenotypic diversity. Such research has found that wing patterning in *Drosophila* is driven by co-option of spatially restricted developmental genes into new roles in pigmentation (Gompel et al. 2005). Studies on body pigmentation in *Drosophila* have unraveled the complexity of cis-regulatory variants and modules that drive different melanic patterns, and how these usually target one of several downstream pigmentation enzymes (Massey and Wittkopp 2016). Complex mimetic wing pattern phenotypes in *Heliconius* butterflies have been found to be regulated by cis-regulatory modifications in the same few developmental genes across mimics (Hines et al. 2011; Reed et al. 2011; Martin et al. 2012; Kronforst and Papa 2015; Nadeau et al. 2016), and inversions have been found to be one way in which mimetic butterflies keep linked variants behind phenotypes intact (Joron et al. 2011). During the last decade, pigmentation genetics research has leveraged expanding genomic resources, new bioinformatic tools, increasingly cheaper high-throughput sequencing, and the availability of modern functional validation techniques to expand beyond a handful of traditional model species to discover underlying routes behind adaptive phenotypes across megadiverse insect taxa.

Bumble bees (Hymenoptera: Apidae: Bombus) exhibit exceptional color diversity, with the approximately 260 species displaying more than 400 color patterns (Williams 2007). Bumble bee color is imparted in their dense setal pile covering their head, thorax, and abdomen. This diversity in color pattern involves multiple colors (e.g., black, orange-red, yellow, white) that are involved in frequent transitions in a sclerite-specific fashion, leading to a wide range of segmental color combinations within and between bumble bee species (Williams 2007; Rapti et al. 2014). One of the primary factors considered to drive this diversity is positive frequency-dependent selection imposed by Müllerian mimicry. The advantages of a shared warning signal have led bumble bees within a geographic region to converge onto similar color patterns, a phenomenon which has resulted in at least 24 distinct regional mimetic color patterns across their global distribution (Williams 2007; Hines and Williams 2012) and has contributed to extensive divergence between and within species. Although the patterns that have come to predominate in a region may be due in part to historical phenotypic frequencies, certain colors appear to be more common in certain climates (Williams 2007). The segment-specific manifestation of color and frequent color transitions across the phylogeny, along with an abundance of natural replicates in bumble bees makes this system well suited for addressing questions in evo-devo and testing fundamental evolutionary hypotheses driving phenotypic diversification (Rapti et al. 2014; Hines and Rahman 2019; Tian et al. 2019). This system is primed for comparative genomic approaches to understanding this color diversity, as it has a well-established phylogenetic framework (Cameron et al. 2007), standardized methods of color pattern quantification (Cameron et al. 2007), multiple high-quality genome assemblies (Sadd et al. 2015; Heraghty et al. 2020; Sun et al. 2020), and detailed developmental staging protocols (Tian and Hines 2018).

**Fig. 1.**
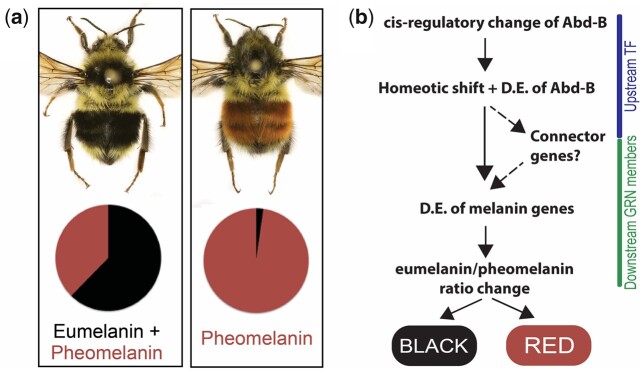
(*a*) Red and black dimorphs of *Bombus melanopygus* where the mimetic color switch occurs in medial (T2+T3) abdominal tergites. The molecular basis underlying the exhibited red versus black switch is achieved by altering the eumelanin–pheomelanin ratio, shown as black versus red respectively in these pie-charts constructed based on data from Hines et al. (2017). (*b*) A proposed molecular mechanism of mimetic color variation in *B. melanopygus*. The upstream regulator *Abd-B* exhibits a spatio-temporal pattern of differential gene expression (D.E.) via a homeotic shift (Tian et al. 2019). This could be directly regulating downstream melanin pathway genes to alter the eumelanin/pheomelanin ratio or could act via additional developmental mediator genes to generate this intraspecies color variation.

In Western North America, bumble bees of multiple species converge onto two prominent mimicry complexes, a form with red abdominal coloration in the Rocky Mountain region, and a form that is largely black in the abdomen in Pacific coastal regions (Ezray et al. 2019). Several species spanning these two regions converge onto local mimicry complexes by switching the coloration of their mid-abdominal tergites from red (Rocky mountain region) to black (Pacific Coastal range). Although most of these polymorphic species exhibit a continuum between their black and red phenotypes across these zones, one species, *Bombus melanopygus*, is dimorphic, showing simple Mendelian inheritance with red being dominant to black (fig. 1*a*). Previous genome-wide association analyses in this species identified a cis-regulatory region of a Hox gene *Abd-B* as the functional locus for this color dimorphism (Tian et al. 2019). Cross-stage gene expression analysis revealed that this posterior abdominal fate-determining gene has undergone a late-developmental heterotopic shift involving upregulation in the more anterior abdominal red segments during setal pigmentation (Tian et al. 2019). Although this research points to a role of a major developmental transcription factor as an upstream switch gene (Tian et al. 2019), how such an upstream regulator generates changes in downstream pigment genes (fig. 1*b*) to generate this color shift remains undiscovered.

The biochemical basis for black and red coloration in vertebrates is a result of two types of melanins, eumelanin and pheomelanin, respectively (Ito and Wakamatsu 2003). Until recently, eumelanin was considered the only melanin type produced in insects (Ito and Wakamatsu 2003). Recent research has revealed that pheomelanin is responsible for red/orange coloration in several insect groups (Galván et al. 2015; Jorge García et al. 2016). Melanin biochemistry research in bumble bee *B. melanopygus* revealed that the black and red colors involve a shift in the ratio of dopamine-derived eumelanin and pheomelanin (Hines et al. 2017; fig. 1*a*). In this case, the black form expresses a combination of black eumelanin and red pheomelanin, with black masking the red, and the red form exhibits only the pheomelanin, allowing red colors to be imparted (Hines et al. 2017). These differences point to a role of the melanin pathway as downstream pigmentation gene targets, however, given the recent discovery of pheomelanins in insects, the enzymes driving pheomelanin production in insects remain largely unknown, being currently inferred from understanding of vertebrate melanin pathways (Galván et al. 2015).

**Fig. 2.**
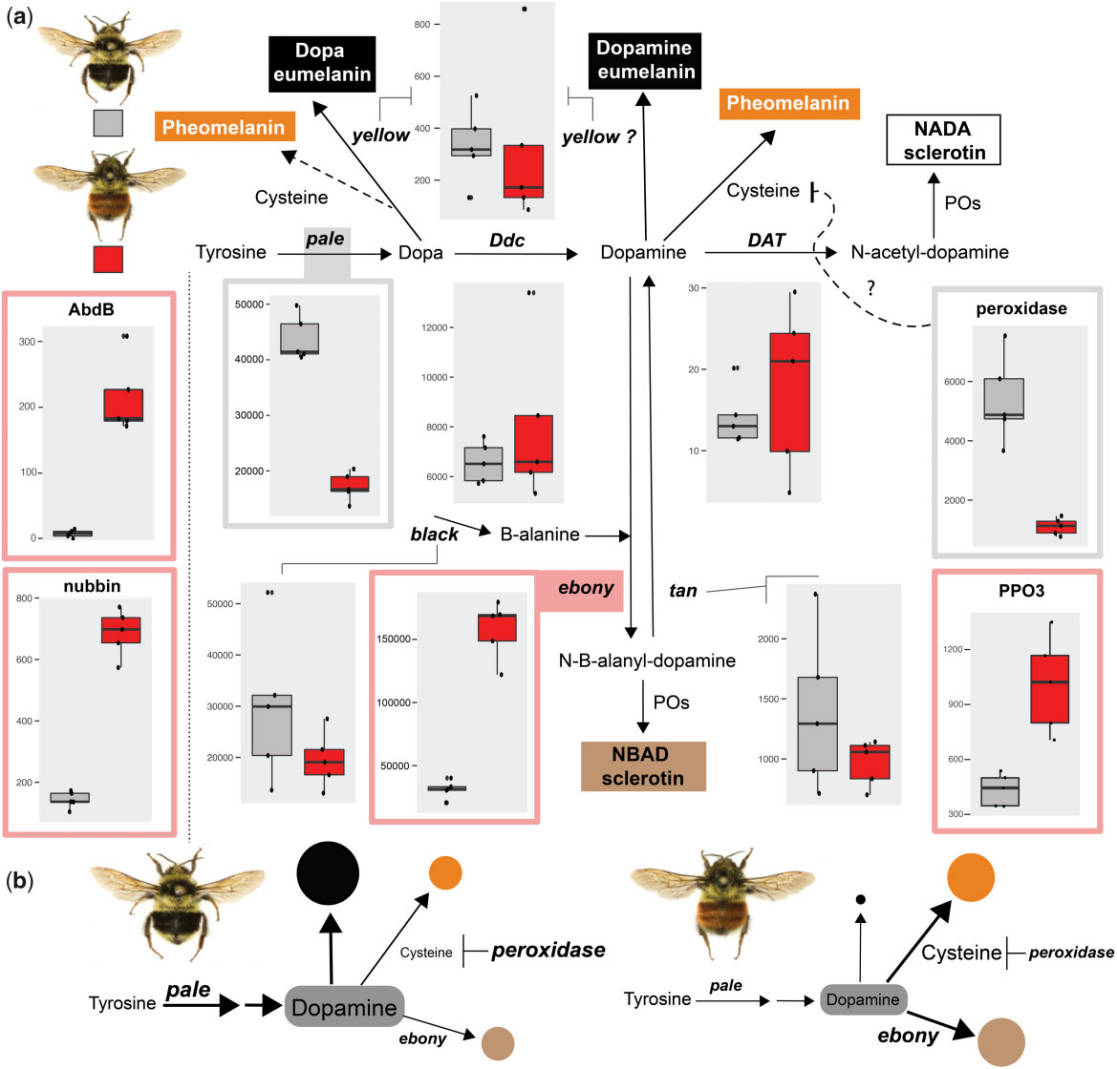
Expression of genes in the melanin pathway inferred from transcriptomic data. (*a*) Onto a simplified schematic diagram of the melanin biosynthesis pathway and their colored end products (adapted from Kuwalekar et al. [2020]; Kronforst et al. [2012]), we provide the expression patterns of each key melanin enzyme for both black and red patterns. The genes which are differentially expressed (FDR adjusted *P* value <0.001) and upregulated in red and black forms are enclosed inside red and gray boxes, respectively. We also show four genes involved in this pathway, either upstream (left, *Abd-B* and *nubbin*) or involved in unknown/multiple locations (right: *peroxidase* and *PPO3*). *Peroxidase* may play a role in inhibiting cysteines. Values on the *y* axis of gene plots are normalized counts and reflect overall amounts of expression of each gene. (*b*) A hypothetical framework for how each color form is generated from changes in the melanin pathway inferred from the gene expression data.

The general schematic (fig. 2) of insect melanin synthesis involves a group of the conserved core set of enzymes (*pale, DDC, DAT, yellow, ebony, tan, black*) each catalyzing a different step in the melanin pathway (Sugumaran and Barek 2016). The repeated deployment of this genetic and biochemical circuitry is consistent across insects (Ferguson et al. 2011; Liu et al. 2016; Qiao et al. 2016; Chen et al. 2019; Zhang et al. 2019). As an initial limiting step in this pathway, L-tyrosine is converted to Dopa via enzyme *tyrosine hydroxylase (pale)* (Sugumaran and Barek 2016). Dopa can then be converted to Dopa-melanin or possibly into pheomelanin if cysteine is present, however, Dopa melanin is rarely produced in insects as it is instead converted to Dopamine using *dopa-decarboxylase (DDC)* (Sugumaran and Barek 2016; Hines et al. 2017). Dopamine is then catalyzed into one of four directions: dark dopamine-melanin likely catalyzed by *yellow*, N-acetyl-dopamine which generates colorless sclerotin by *DAT*, N-ß-alanyl dopamine (NBAD) to produce light yellow-brown NBAD sclerotin via *ebony* and necessary precursors from *black* (this effect can be countered by the gene *tan*, which converts this back to dopamine) (fig. 2), and finally, it is thought that dopamine will naturally convert to pheomelanin without needing an enzyme as long as cysteines are present (Meiser et al. 2013; Sugumaran and Barek 2016). Multiple research studies have examined the molecular targets of light to dark pigmentation differences across several *Drosophila* species (Gompel et al. 2005; Jeong et al. 2008). Together these have revealed that many of these melanin enzymes are targeted, especially *yellow*, *ebony*, *tan*, and *pale* (Kronforst et al. 2012), and that these targets typically occur in cis-regulatory regions (Rebeiz and Williams 2017), thus altering tissue-specific expression of these pigment enzymes. Expanding pigmentation genetic research beyond *Drosophila* allows us to test the degree of molecular-level convergence in the production of similar phenotypic outcomes across megadiverse insect taxa and ultimately helps us to understand how a conserved gene regulatory network can be tweaked in numerous ways to drive phenotypic diversification.

This study aims to investigate the key downstream players generating the mimetic color dimorphism in the bumble bee *B. melanopygus*. Leveraging the recent progress on developmental staging (Tian and Hines 2018), melanin biochemistry (Hines et al. 2017), and color genetics (Tian et al. 2019) in bumble bees, we perform a transcriptomic analysis on a critical setal developmental stage to look for key genes associated with setal color differentiation. We then examine temporal expression patterns of candidate genes across critical color differentiation stages using quantitative real-time PCR (q-PCR) to assess their association with the color switch. Overall, our research identifies key players of the gene regulatory cascade underlying the melanic color dimorphism in *B. melanopygus*, providing insights into regulatory mechanisms underlying pigmentation and establishing a suite of candidate gene targets capable of generating mimetic phenotypes across this color-diverse group.

## Results

### Color Morph-specific Gene Expression Pattern

**Fig. 3.**
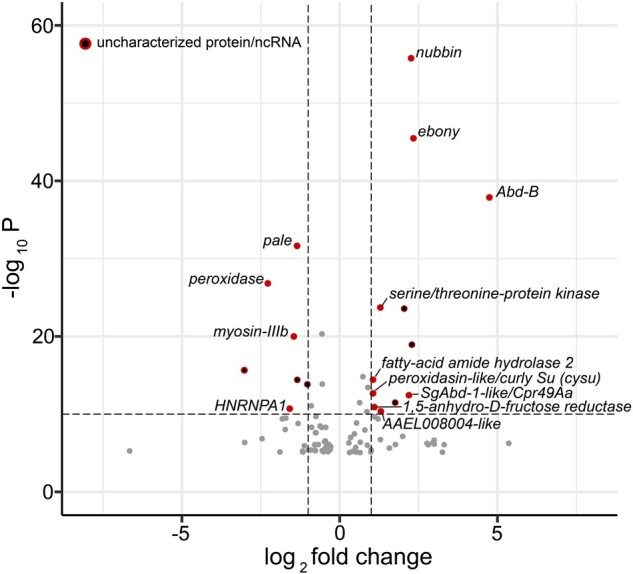
A volcano plot of all (*n* = 95) differentially expressed genes (DEGs) which passed the significance threshold (FDR adjusted *P* value <0.001). The upper right-most and upper left-most rectangles of the plot exhibit the most upregulated genes in the red and the black form, respectively. The most significant genes (*P* value ≤10^−10^) with higher log_2_ fold changes (−1 ≥ log_2_FC ≥ 1) are represented with solid red dots and are annotated either using *Bombus impatiens* gene annotation or orthologous gene sequence information from *Drosophila melanogaster*. Six genes that passed the aforementioned criteria are “uncharacterized proteins/ncRNAs” and are represented using a red circle with a black center.

Our transcriptomic analysis was performed on black and red epidermal tissues of the color polymorphic second and third metasomal segments (five bee replicates/morph, supplementary table 1, Supplementary Material online), dissected at the point of adult eclosion (0-h callow stage), the stage with peak differential expression (10–20× difference) between color forms in *Abd-B* (Tian et al. 2019), and in which color differences start to become readily apparent. This thus should enable implicated genes from most upstream (*Abd-B*) to downstream pigment genes to be identified. We identified 95 differentially expressed genes (statistically significant at FDR-adjusted *P* value <0.001). Among these, 44 and 51 genes were upregulated in red and black morphs, respectively. The gene list and their *Drosophila melanogaster* and *Bombus impatiens* orthologs are provided in supplementary table 2, Supplementary Material online. Among the red form-biased genes, *nubbin (nub)*, a key developmental transcription factor in the fruit fly *D. melanogaster* (fig. 3 and supplementary table 2, Supplementary Material online), and *ebony (e)*, a core melanin pathway gene, are the top and second most upregulated genes, respectively. Following these genes is *Abdominal-B* (*Abd-B*) as the third most upregulated gene in red phenotypes. Other significantly upregulated genes include *serine/threonine protein kinase, fatty acid amide hydrolase 2*, and *peroxidasin-like* (orthologous to *D. melanogaster Curly Su* [*cysu*]). Among the black morph-biased genes, *pale*, *peroxidase*, *myosin-IIB*, and *Heterogeneous Nuclear Ribonucleoprotein A1* (*HNRNPA1)* (fig. 3 and supplementary table 2, Supplementary Material online) are the top differentially expressed genes. We also discovered many genes of uncharacterized function in both color morphs, which include several (*n* = 7) ncRNAs, some of which were ranked high on this list (supplementary table 2, Supplementary Material online).

### Identification and Annotation of Melanin Pathway Candidate Genes

Figure 2 shows the expression patterns of the central melanin pathway genes inferred from the transcriptome data. Two genes in this pathway are modified in response to upstream regulators, whereas several other genes are unaffected. Specifically, *ebony* and *pale* have high expression and marked differential expression patterns, being upregulated in red and black forms, respectively. Other major melanin pathway genes were not differentially expressed and were expressed either in low (i.e., *DAT*), moderate (i.e., *yellow*, *tan*), or high amounts (i.e., *Ddc*, *black*) in both forms. We also found several genes that are differentially expressed that are involved in oxidation reactions, and which may act in the generalized “phenoloxidase” steps known to occur throughout various parts of the pathway to catalyze melanin reactions. These include *PPO3*, *peroxidase,* and *Curly Su (cysu)*. These each have different relative patterns of expression, thus may be playing roles in different parts of the pathway to help regulate pigment balance. A hierarchically clustered heatmap of all differentially expressed genes (supplementary fig. 1, Supplementary Material online) shows the most similar expression of *ebony* with *nubbin*, *Abdominal-B (Abd-B)*, *Fatty acid amide hydrolase* (CG8839), CG133314 (expressed exclusively in late pupal stages in *Drosophila*, no known annotation/function), CG15020 (associated with chitin-based cuticle formation, mitogen-activated protein kinase which has known role in melanogenesis), and a key developmental gene, *disc large 1* (*dlg1*). On the other hand, *pale*, the most upregulated gene in the black form, is clustered with antioxidant enzyme *peroxidase*, CG31974 (no known annotation/function), a toll pathway gene, *toll* (*Tl*) (toll genes have relationships with melanin with regard to immunity), and cell adhesion gene *cadherin 88C* (*Cad88C*) (Maître and Heisenberg 2013).

### Gene Ontology Enrichment Analysis 

**Fig. 4.**
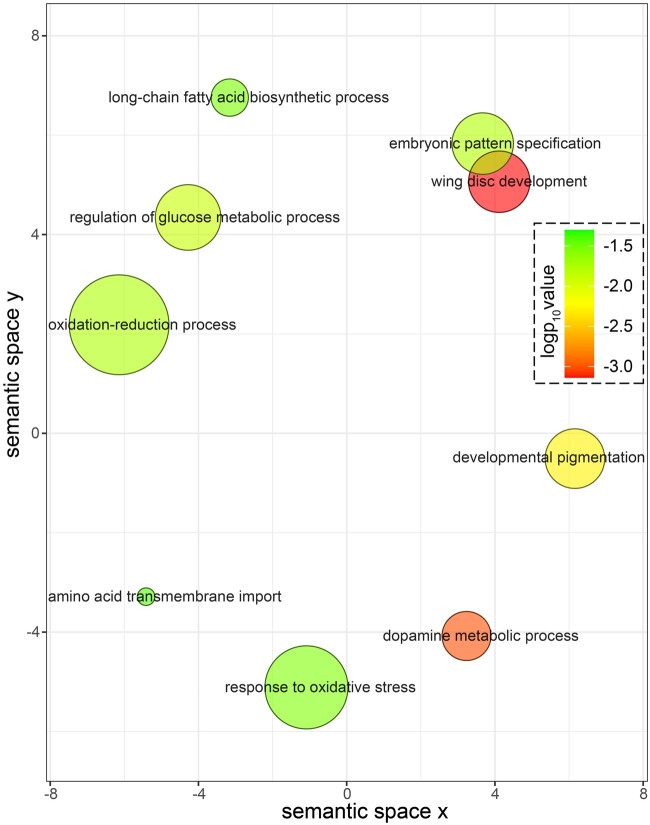
A scatter plot of the summarized enriched gene ontology (GO) clusters related to biological processes obtained from ReviGO web server. Each individual bubble represents one semantically simplified functional cluster and bubble sizes are based on the log value of the term sizes. Bubble colors indicate the log_10_(*P* value) ranging from maximum *P* value of 0.05 (green) to lower values.

Gene ontology (GO) enrichment analysis identified 43 statistically significant (*P* < 0.05) terms (supplementary table 3, Supplementary Material online) from multiple functional annotation sources (GO biological processes, molecular function cellular component; Interpro, UniProt Keywords, and sequence features) that were enriched in the differentially expressed gene set. Examining GO biological process terms (*n* = 10) revealed significant overrepresentation of terms associated with pigmentation-related activities including dopamine metabolic process, developmental pigmentation, oxidation–reduction process, adult chitin-containing cuticle pigmentation, and response to oxidative stress. We also observed enrichment of two terms associated with developmental processes including wing disc development and embryonic pattern specification. A simplified visual representation of GO terms associated with underlying biological processes performed in ReviGO supported nine major clusters (fig. 4) with wing disc development, dopamine metabolic process, and developmental pigmentation being the most significant ones. Other prominent clusters were response to oxidative stress, fatty acid biosynthesis, glucose metabolism, and transmembrane transport of amino acids. Major enriched GO terms associated with molecular functions involved heme binding, peroxidase activity, and iron ion binding activities. Enriched GO terms associated with cellular components were primarily membrane related, indicating a high activity of transmembrane transport occurring during this developmental stage.

### Expression Pattern of Candidate Genes across Developmental Stages

**Fig. 5.**
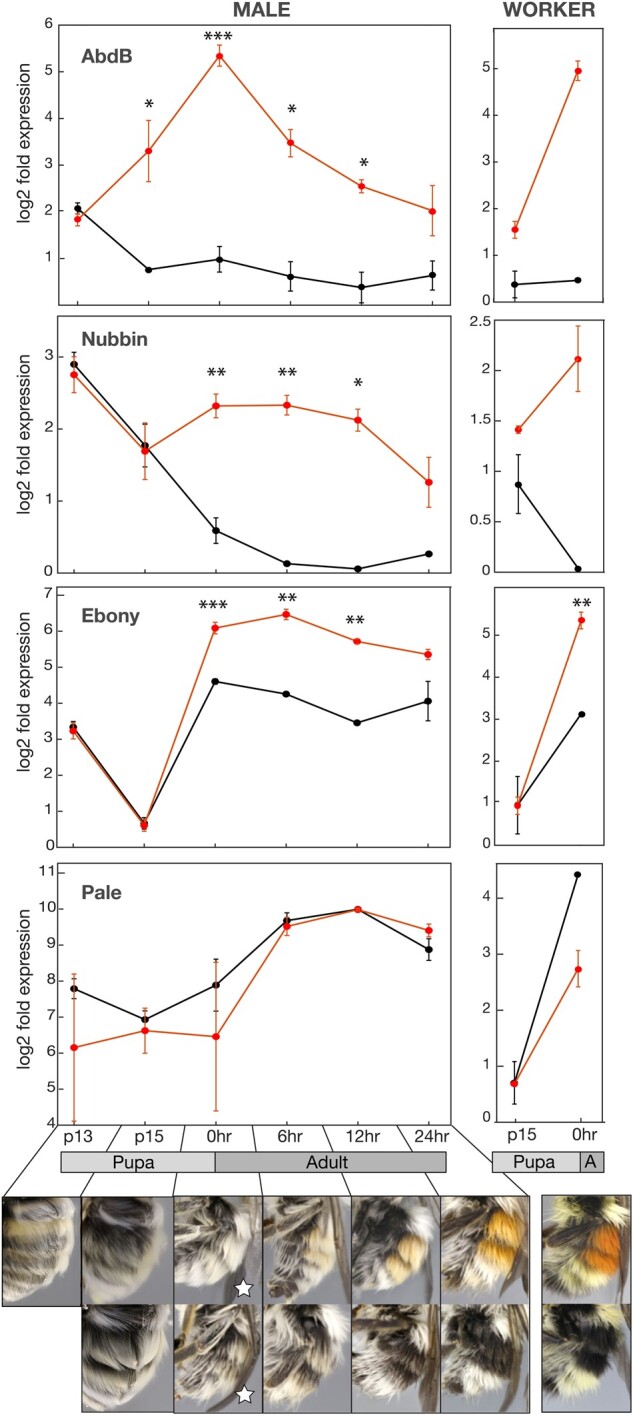
Gene expression of red and black form T2+T3 epidermal tissues from throughout late pupal and early adult development inferred using quantitative PCR. Depicted are averages and one SE from multiple samples from each stage for red (red line) and black (black line) male and worker *Bombus melanopygus*. Photos show the coloration of setae at each stage from P13—24 h as well as the final adult coloration. P13 is the same between both stages thus one photo, taken from *Bombus impatiens*, is shown. White stars indicate the stage used for transcriptomic sampling. Asterisks indicate significance with *P = 0.01–0.05, **P = 0.001–0.01, and ***P < 0.001. The panel at the bottom includes the same original photos used in Tian et al. (2019, figure 3) for P15 through adult stages, and same original photo used in Tian and Hines (2018, figure 6) for P13.

In the cross-developmental qPCR data, the first gene analyzed to be differentially expressed is *Abd-B*. In *Abd-B*, the red morph expresses the same amount as the black morph in pupal stage P13, after which the red dramatically increases expression above the black, retaining divergent expression patterns from pupal stage P15 through to the 24-h adult period (Significant from P15 to 12-h adult, supplementary table 4, Supplementary Material online, thus throughout pigment deposition; fig. 5). Expression differences peak at the 0-h adult stage. For both *ebony* and *nubbin*, differential expression in males starts at 0-h callow adults, with the red morph displaying much higher levels compared with the black morph (fig. 5). This differential expression is maintained throughout pigment deposition at similarly high levels but peak divergence is at 6 and 12 h, a bit later than *Abd-B*, and when pigment change is greatest (fig. 5). Workers follow the same pattern as males, but *nubbin* is somewhat divergent in P15 similar to *Abd-B*, suggesting that differences in *nubbin* may proceed that of *ebony* somewhat in timing. In males, the *pale* gene exhibited no clear differential expression and high variance, with some indication that there may be higher expression for the black form around 0 h only, but this is not statistically significant (fig. 5). In workers *pale* expression suggests more expression in the black form at 0 h, similar to the transcriptomic data, although this is not statistically significant. *pale* may have a narrower window of differential expression that was missed with these data. Higher expression in pupal stage P13 than P15 for *ebony* could be due in part to increased expression in the main cuticle which is not fully melanized at P13, whereas by P15 this is fully melanized and the setae begin to show the first differences in melanization (fig. 5). This decoupling of the timing of primary cuticle and setal melanization makes it possible to separate genes related to the melanization of the primary cuticle from those involved in the melanization of setae.

## Discussion

This study utilized a developmental transcriptomics approach to put together multiple components of the gene network underlying the mimetic color pattern switch in *B. melanopygus*. Our differentially expressed gene list included genes at all levels, from upstream developmental switch genes to downstream enzymes directly involved in melanin synthesis. This gene set included dramatic upregulation of the upstream Hox gene *Abd-B* in the red morphs during callow stages, consistent with previous results (Tian et al. 2019, figure 5). It has also identified several downstream genes in the melanin pathway involved in this phenotype, namely *ebony* in the red form, and *pale* in the black form. These data suggest a plausible explanation for how the melanin pathway can be tweaked to alter the eumelanin versus pheomelanin ratio that generates the two distinct color forms.

The generation of red pigments depends on the preferential production of pheomelanin over eumelanin. Melanin pigments are primarily dopamine-derived in *B. melanopygus* (Hines et al. 2017) and other insects (Liu et al. 2014; Chen et al. 2019). Given the melanin pathway (fig. 2), we would expect that there are two major routes to controlling melanization: altering how much dopamine gets produced through altering tyrosine conversion via *pale*, and altering how that dopamine gets processed. As outlined previously, dopamine can be converted into clear NADA sclerotin, yellow NBAD sclerotin, dark eumelanin, and red pheomelanin depending on the enzyme present. In processing dopamine, pheomelanin synthesis is kinetically favored above eumelanin and sclerotin production, as pheomelanin can be produced without the need for an enzyme as long as the necessary cysteine is present (Ito 2003). Our data support the altering of pheomelanin to eumelanin ratios through both of these routes. In the red morphs, reduced dopa production via downregulation of *pale* prevents excessive dopamine production. Keeping dopamine levels low ensures that the dopamine does not occur in excess of the amounts of free cysteines present, thus enabling only pheomelanins to be produced. As a reinforcement mechanism, *ebony* upregulation in the red forms also keeps dopamine low and instead generates pale “tan” sclerotins, which would fail to mask red color. In black morphs, the reversal of expression pattern of these two melanin genes (i.e., *pale* and *ebony*) ensures ample production of dopamine to exhaust the cysteines to initially produce some pheomelanin, followed by a substantial amount of eumelanin, resulting in the black setal color. Switching between melanin types using *ebony* has been documented to occur in *Drosophila*, which uses this to switch between dark and light melanins (Wittkopp et al. 2002; Massey and Wittkopp 2016). The masking effects of eumelanins on other pigments to generate patterns has been observed in other insects, such as butterflies (Martin et al. 2012) and *Oncopeltus* bugs (Liu et al. 2014, 2016).

Redox reactions are essential components of melanization, which involves oxidation processes for final catalysis of pigments (Napolitano et al. 2013). The upregulation of *peroxidase (pxd)*, a redox gene in the black form, supports its roles in the melanogenic pathway (Prota 1992; García-Molina et al. 2005). In *Drosophila*, *pxd* is known to convert antioxidant glutathione from its active (reduced) form to its inactive (oxidized) form, glutathione disulfide. Glutathione is primarily formed of three amino acids, including cysteine, and it is required to be present in its active form to supply readily available cysteines needed for pheomelanin production (Sugumaran and Barek 2016). Therefore, high *pxd* expression may inhibit cysteine production, resulting in reduced pheomelanin synthesis in the black form. This may thus act as a third reinforcement mechanism for generating high levels of eumelanin required for black pigments. We have also discovered upregulation of a peroxidasin gene, *Curly Su*, in the red morphs. *Curly Su* is involved in the *Drosophila* wing maturation process in the posteclosion stage and has peroxidase activity (Bailey et al. 2017). Although both genes likely play roles in redox reactions involved in melanization, which is an oxidation process, their opposing expression patterns may indicate that they play counteracting roles.

Several other genes (e.g., cytochrome P450s, *dual oxidase* [*Duox*], *prophenoloxidase 3* [*PPO3*]) with known roles in innate immunity defense, oxidation–reduction reactions, and in response to oxidative stress (Scott and Wen 2001; González-Santoyo and Córdoba-Aguilar 2012; Lu et al. 2014; Lee et al. 2015) were found in our differentially expressed gene list (supplementary table 2, Supplementary Material online). In vertebrates, dopamine-derived biosynthesis of pheomelanin relies on the utilization of antioxidant glutathione which can expose the implicated cellular machinery to a high level of oxidative stress and subsequent mutagenic effects during pheomelanin-rich anatomical structure development (Roulin et al. 2011; Napolitano et al. 2014; Galván and Solano 2015). Red color morphs of *B. melanopygus* converge onto their local mimicry ring distributed across high altitudes of Rocky mountain ranges (Ezray et al. 2019), which could further predispose them to higher levels of oxidative stress. These other genes may perform roles in countering some of this stress.

GO analysis results (fig. 4 and supplementary table 3, Supplementary Material online) show unexpected enrichment of two biological processes: embryonic pattern specification and wing disc development. The enrichment of embryonic pattern specification may reflect the observed co-option of major early developmental genes toward driving these late developmental phenotypes, such as what is observed with the co-option of *Abd-B*. Genes *toll*, *pipe*, and *drumstick*, upregulated in the black form (supplementary table 2, Supplementary Material online), comprised the GO term embryonic pattern specification. *Toll* and *pipe* are part of the toll signaling pathway genes and also have roles in immune response (Hashimoto et al. 1988; Anthoney et al. 2018), an alternative function of melanization in insects. We have also discovered the upregulation of *tollo* (Toll-like receptor) in the red form, which has multiple roles in innate immune response (Akhouayri et al. 2011) and developmental processes (Anthoney et al. 2018). The involvement of *toll* signaling pathway genes has previously been found in sex-specific mimetic wing coloration in swallowtail butterflies (Nishikawa et al. 2013).

One of the genes involved in wing disc development that is also an early embryonic gene is *nubbin*. *nubbin* had clear differences in expression being the top upregulated gene in the red color morph. Its differential gene expression pattern starts after *Abd-B* and before the pigment enzymes, raising the possibility that *nubbin* could work as a developmental mediator gene between upstream regulator *Abd-B* and downstream players. In *D. melanogaster*, *nubbin* expression is highest in the early embryonic stages, whereas low levels of expression of *nubbin* are observed in the late pupal stages (Brown et al. 2014). In *Drosophila*, as well as in other organisms, *nubbin* has traditionally been recognized as a key selector gene in regulating the proximal–distal patterning process during wing development (Ng et al. 1995), with some researchers purporting the presence of *nubbin* to indicate homology of a structure with wings (Prud’homme et al. 2011). In *Drosophila*, however, additional roles in embryonic cell differentiation and migration (Anderson et al. 1995), neurogenesis (Billin et al. 1991), and invagination of spiracles (Hu and Castelli-Gair 1999) have been found and analysis of *nubbin* expression domains and functional roles in other arthropods (Averof and Cohen 1997; Abzhanov and Kaufman 2000; Damen et al. 2002; Gibert et al. 2002; Li and Popadić 2004) revealed that *nubbin* plays diverse roles in development beyond its well-characterized activity as a “wing” gene during early embryonic development. For example, it evolves at a higher pace than other homeotic genes and is a critical developmental transcription factor involved in limb development (Li and Popadić 2004; Hrycaj et al. 2008; Turchyn et al. 2011).

There is no known interaction of *nubbin* with melanin pathway genes. However, *nubbin* has known interactions with *Hox* genes. It is responsible for suppressing limb development by regulating *abd-A* activity in abdominal segments of *Oncopeltus fasciatus* (Hrycaj et al. 2008). In *Drosophila*, *Abd-B* orchestrates posterior spiracle development, and *nubbin* is one of the five transcription factors (i.e., *cut*, *nubbin*, *empty spiracles*, *spalt*, and *Klumpfuss*) positioned in the immediate downstream of the underlying gene regulatory cascade (Hu and Castelli-Gair 1999). Some of these *Abd-B* responsive genes are known developmental regulators of pigmentation, including *spalt*, which has an established role as a critical regulator of eyespot color formation in butterflies (Zhang and Reed 2016), and *cut* which was implicated in yellow color variation in bumble bee *B. terrestris* (Rahman et al. 2020). Taken together, our research suggests a novel upstream player in pigmentation, showing a likely role of *nubbin* in late pupal development for driving pigmentation phenotypes. It supports the argument that *nubbin* is evolutionarily labile and frequently co-opted across insect orders and thus should not be labeled a “wing” gene (Mikó et al. 2012). Additional research is required to determine how *nubbin* could be deployed and integrated as a part of the genetic circuitry underlying melanin biosynthesis in bumble bees.

In this study, following a developmental transcriptomic approach has enabled us to assemble components of a gene network, from the established most upstream molecular target (i.e., *Abd-B*), through potential developmental intermediator genes (e.g., *nubbin*), to downstream pigmentation genes (i.e., *ebony* and *pale*). We have shown that several complementary mechanisms involving multiple pigment genes can act in concert to alter the melanic fate that ultimately drives this red versus black color switch. *Drosophila* research has found a few upstream players in abdominal patterning (e.g., *bab*, *Abd-B*), but mostly has found that the initial genetic targets lie in cis-regulatory regions of specific pigment genes. In this system, we see that some of the most upstream and early developmental genes can be co-opted into these new roles and that downstream genes need not be direct targets. Upstream genes may even facilitate the more complex changes in downstream pigment genes observed.

Several other comimicking bumble bee species (e.g., *Bombus bifarius*, *Bombus sylvicola*, *Bombus flavifrons*) exhibit the same red versus black abdominal color shift as *B. melanopygus*, and red-black color shifts are involved in both intraspecific and interspecific color divergence across the highly color-diverse bumble bees. These data provide a foundation for future research examining mechanisms underlying the radiation in coloration and extensive convergence in this system. With these genes in hand, the multiple routes to the same phenotype can be determined: whether the same gene, other members of this network, or entirely different genes and gene networks, are altered in each convergent acquisition of mimetic phenotypes.

## Materials and Methods

### Bee Colony Rearing and Stage-Specific Pupa Dissection

Field collected *B. melanopygus* queens were allowed to initiate colonies under laboratory conditions (28 °C, 60% RH, complete darkness). For transcriptome sequencing, we used dissected tissue from newly emerged male bees (also known as 0 h callows) of both red and black forms, five replicates (bees) for each color form. These were drawn from three colonies for red form and two colonies for black form individuals. 0-h callows were sampled at the point in which a bee fully emerges from its pupal brood cell and were collected between 0 and 1 h old (Tian and Hines 2018). Phenotypes were already partially apparent in the collected segments in all bees. As red coloration is the dominant phenotype, thus being displayed by both homozygous and heterozygous individuals, we confirmed individuals sampled were from colonies that are homozygous through observing queen (red only) and offspring (red males and workers produced only) phenotypes and genotyping several workers of their host colony by PCR amplification and sequencing at the previously identified color locus (primers 1F/1R, see Methods in Tian et al. [2019]). Heterozygotes are apparent by double peak patterns in resulting sequencing chromatograms.

Epidermal tissues from the second and third metasomal segments were dissected. After removal of the thorax and head, the abdomen was bissected longitudinally and the gut contents, heart, and sternites with attached tissue were removed. The sample was then immersed in ice-cold PBS buffer where the second and third tergites were separated from the remainder. These tergites were cleaned to remove trachea and muscles that were attached until just the epidermis remained, still attached to the cuticle (setal cells are broken if the epidermis is separated from cuticle). These were placed in an Eppendorf tube and flash frozen on dry ice, followed by storage at −80 °C until extraction.

### RNA Extraction and Sequencing

Dissected tergites were homogenized in their tubes for 35 s on low in Trizol buffer using the Omnibead ruptor, with four metal beads, followed by extraction using standard protocols in the Direct-zol RNA Miniprep Plus kit, including the DNase I removal step. Sufficient RNA quality and quantity were confirmed using an Agilent Bioanalyzer, followed by preparation for Illumina sequencing using a TruSeq Stranded mRNA Kit (Illumina). Multiplexed libraries were sequenced at 75-bp paired-end using the Illumina NextSeq 550 at the Penn State Genomics Core Facility (University Park, PA), with a coverage aimed at generating approximately 16 million reads for each mRNA library (ten samples were run together alongside 15 other Hymenopteran and pollen RNA samples for other studies; 18–22 million raw reads obtained/sample; supplementary table 1, Supplementary Material online).

### Differential Gene Expression Analysis

Quality assessment of the raw paired-end reads was conducted using FastQC v0.11.9 (Andrews 2010). To retain only high-quality reads and discard any reads less than a minimum length (36 bp), the raw reads were trimmed using Trimmomatic v. 0.39 (Bolger et al. 2014) with the following parameters: SLIDINGWINDOW:4:30 ILLUMINACLIP:adapters.fa:2:30:5 MINLEN:36 LEADING:3 TRAILING:3. Posttrimming reads were further assessed for quality using FastQC v0.11.9 (Andrews 2010). Properly paired trimmed reads were utilized for an alignment-based gene-level differential expression analysis of the five biological replicates each for black versus red phenotypes. For this, trimmed reads were aligned to the *B. impatiens* genome (RefSeq GCF_000188095.3, BIMP_2.2) (Sadd et al. 2015) with a splice-aware aligner HISAT2 v.2.2.1 (Kim et al. 2015) using default parameters followed by postprocessing (i.e., sorting and indexing) of the alignment files using Samtools v.1.3 (Li et al. 2009). Sample information, number of raw reads, posttrimming reads, and alignment statistics are reported in supplementary table 1, Supplementary Material online. Raw transcriptomic sequencing reads are available from NCBI SRA archive under NCBI BioProject PRJNA721780.

To estimate per sample gene-level abundance, featureCounts v.2.0.1 (Liao et al. 2014) was used to count reads aligned to specific genomic intervals from each sample-specific alignment file, leveraging the gene structure information (GCF_000188095.3_BIMP_2.2_genomic.gtf) provided with the *B. impatiens* reference genome assembly (Sadd et al. 2015). Per-sample raw count data for each genomic location were extracted for differential gene expression analysis, which were performed using the DESeq2 R package (Love et al. 2014) (the utilized R script is available from Dryad data repository doi.org/10.5061/dryad.pk0p2ngnf). A volcano plot comparing fold expression to *P* value of support for differential expression was obtained using the “EnhancedVolcano” R package (Blighe et al. 2019). Normalized counts obtained using DESeq2 (Love et al. 2014) were utilized to obtain a hierarchical clustered heatmap using heatmap.2 function of the gplots R package (Warnes et al. 2009). *Drosophila melanogaster* homologs of differentially expressed genes were identified using the BioMart utility (Kinsella et al. 2011) from Ensembl database release 48 (Howe et al. 2019) and FlyBase v. FB2020_04 (Thurmond et al. 2019) orthologous gene information (supplementary table 2, Supplementary Material online) and were compared with the annotations obtained from *B. impatiens* (Sadd et al. 2015).

### Identification and Annotation of Melanin Pathway Candidate Genes

To more closely examine expression patterns in our transcriptomes of known melanin pathway candidate genes, we used TBlastX (TBlastX program parameters: -outfmt 6 -evalue 1e-8 -max_target_seqs 1 -max_hsps 1) of the NCBI BLAST+ command line utility (Camacho et al. 2009) to locate *D. melanogaster* transcripts of seven genes that have a well-established role in the melanin pathway (*ebony, pale, black, DAT, DDT, tan, yellow*), along with three other genes of interest from our differentially expressed genes including *nubbin* and two genes likely part of the melanin pathway—*prophenoloxidase 3, peroxidase—*in the *B. impatiens* transcriptome (Sadd et al. 2015). Normalized read counts for these identified genes were used to build Tukey-style box plots using R package ggplot2 (Wickham 2011) representing normalized count data.

### Gene Ontology Enrichment Analysis

Gene ontology enrichment analysis (Huang et al. 2009) was performed using the Database for Annotation, Visualization and Integrated Discovery (DAVID v. 6.8) (Dennis et al. 2003). Unique FlyBase IDs (*n* = 66) of *D. melanogaster* orthologs of identified differentially expressed genes served as the query gene list. A list of all unique FlyBase IDs (*n* = 7,083) representing all *B. impatiens* transcripts was obtained through BLAST by searching against the *D. melanogaster* transcriptome (TBlastX program parameters: -evalue 0.000001 -outfmt 6 -max_target_seqs 1 -max_hsps 1) and was amalgamated with a few additional manually curated unique FlyBase IDs from the aforementioned differentially expressed gene set to generate the final background gene list of unique FlyBase IDs (*n* = 7,092). As we are primarily interested in exploring the functions of the most differentially expressed and most reliable set of genes, we identified GO terms which met the EASE score (Hosack et al. 2003) from the set of genes that passed an adjusted Fisher’s exact test *P* value significance level threshold of *P* < 0.05 and an FDR-corrected significance threshold of *P* < 0.001. To summarize the GO terms, we used ReViGO web-based interactive server (Supek et al. 2011) which uses a multidimensional scaling (MDS) approach to cluster GO terms based on semantic similarity measurements. GO terms related with biological processes (*n* = 18) and their corresponding *P* values obtained from the aforementioned procedure (i.e., using DAVID v. 6.8) were provided as the input with default settings in the ReViGO interface (available at: http://revigo.irb.hr/) to generate a scatter plot depicting the major nonredundant biological process clusters.

### q-PCR Validation of Candidate Genes

To assess the developmental trajectory of targeted genes of interest including *Abd-B*, *nubbin*, *ebony*, and *pale*, we performed RT–PCR (q-PCR) on RNA extracted from T2+T3 epidermal tissues from both red and black form male bees from across several developmental stages during, before, and after the 0-h time point used for transcriptomics. We performed qPCR on these new genes using the same RNA previously extracted to perform qPCRs of *Abd-B* in Tian et al. (2019), including individuals from pupal stages P13 and P15, as well as 0, 6, 12, and 24 h callows. These stages were chosen because the gene driving these differences in expression, *Abd-B*, begins to be differentially expressed at P15, and melanic pigmentation is nearly complete by 24 h (*Abd-B* expression also is more similar between both at this time point; Tian et al. 2019) thus these time points straddle the most important stages for gene expression differences. Most biological treatments were sampled at three to four replicates each, although for a few there were only two replicates (see supplementary table 5, Supplementary Material online, for sample distribution). This previous study extracted T2 and T3 segmental RNA separately from a different set of red and black colonies than used for our transcriptomics. We combined equimolar amounts (as inferred via Thermo Scientific NanoDrop One) of T2 and T3 RNA to obtain a pooled T2 and T3 sample, performed a new cDNA synthesis, and performed quantitative PCR using SybrGreen and technical replicates in triplicate, following the same protocols as outlined Tian et al. 2019. We optimized qPCR primers to span exons, not span exons known to be involved in alternative splicing, include regions with sequence conservation between color forms (determined by comparing the assembled genomes from Tian et al. [2019]), and not yield primer dimers. Each primer set was found to have an approximate efficiency of doubling every CT cycle, as inferred using a standard curve run with five dilutions at 5× differences. This included the following primers: *nubbin* (F: 5′-CGTAACCACTCCCGATCACA-3′; R: 5′-GTCCATCCACCTCACCATGA-3′; 149 bp fragment spanning exons 3 and 5 spaced ∼51 kb apart), *ebony* (F: 5′-TCTGTTGACCAATCGGAAGC-3′, R: 5′-TACCAGCCGCATCTCTTTGT-3′; 106 bp insert spanning exons 7 and 8 spaced 234 bp apart), and *pale* (F: 5′-GGATCGTACCTCACCTCGAA-3′, R: 5′-TCTATTACGTGGCGGAAAGC-3′; 97 bp insert spanning exons 1 and 2 spaced 264 bp part). The splice junctions and fragment length of these primers in *B. melanopygus* were validated through PCR and sequencing on cDNA of an exemplar specimen (0 h, 136-2 Black form). Each gene was run on a separate plate along with a control gene, v*ATPase*, which was determined to be optimal among a set of control genes in Tian et al. (2019). *Pale* was run twice because of high observed biological variance, but yielded similar results both times, so the trial with lowest technical replicate variance was reported. *Abd-B* data were obtained from CT results from Tian et al. (2019), averaging the normalized values obtained for T2 and T3, which were normalized against the same control gene sample on the same plate and are thus comparable. We utilized *Abd-B* CT data for this from the same individuals used for the other genes (thus some of the samples from Tian et al. [2019] were excluded) to enable direct comparisons between genes.

In addition, to compare similarities between males and female workers, we utilized the same techniques above on worker tissues from stages P15 and 0 h, using RNA obtained by Tian et al. (2019) (supplementary table 5, Supplementary Material online). In this case, 0-h black and P13 black each had only one replicate, 0 h red had two replicates, and the rest had three replicates. All worker samples were run on a single plate.

Given the optimal efficiency of primers, we did not normalize the resulting CT values using a standard curve, but rather normalized gene expression against the v*ATPase* value (subtracted the difference in CT for *vATPase* from the lowest level [highest CT] of expression and added this number to the test gene CT). *vATPase* raw CT values were very close within each stage across samples of both color forms (<1 CT difference, usually <0.5 CT), however, there were some differences in average *vATPase* values by stage that were consistent across genes (e.g., for *ebony*, average CT for P13:19.5, P15:18.5, 0 h: 17.9, 6 h: 17.7, 12 h: 17.5, 24 h: 17.8) that may impact interpretation of patterns between stages (P13 could appear ∼1 value higher that P15 if this is driven by differences in *vATPase* in development). The resulting values were then normalized to represent log_2_ fold difference expression by setting a value rounded up from the lowest expression (highest CT) at zero within each gene and sex/caste combination and adjusting the other values accordingly by subtracting from this CT value the normalized CT values. For *Abd-B*, averaged values from Tian et al. (2019) were converted to log_2_ fold difference from the lowest expression value in the same manner. Given that fold normalization from zero was calibrated within each gene/sex, the values obtained should be roughly comparable across genes and conditions in relative fold expression differences among treatments, but not comparable in the absolute fold number on the *y* axis. We tested the statistical significance between stage-specific black and red form for each gene in log_2_ fold difference data using Welch’s two-sample *t*-test, which accounts for unequal variances. In 0-h worker stages, we used two-sample *t*-tests as we have only one replicate for black forms.

## Supplementary Material

Supplementary data are available at *Genome Biology and Evolution* online.

## Supplementary Material

evab080_supplementary_Data
