# Supplementary material for: Developmental Transcriptomics Reveals a Gene Network Driving Mimetic Color Variation in a Bumble Bee
Source: Genome Biol Evol. 2021 Apr 21;13(6):evab080. doi: 10.1093/gbe/evab080 (PMC8220310; doi:10.1093/gbe/evab080)
Supplement: evab080_supplementary_Data [file evab080_supplementary_data.zip › Supplementary Information_GBE_final.docx]

**Supplementary Information**

**Supplementary Table 1:** A table reporting the summary statistics for transcriptomic samples utilized in the RNA-Seq experiment. Source colony (2 for black, 3 for red) is indicated by the first identifier in the sample name.

| Sample name | Phenotype | | NCBI SRA Accession | Raw read pairs (in millions) | Post-trimming  read pairs  (in millions) | Alignment rate to reference  (BIMP 2.2) |
| --- | --- | --- | --- | --- | --- | --- |
| 10B_1_S18 | Black | SRR14234413 | | 19.38 | 13.48 | 88.03% |
| 10B_2_S19 | Black | SRR14234412 | | 18.44 | 12.72 | 88.36% |
| 10B_3_S20 | Black | SRR14234411 | | 18.26 | 12.71 | 88.72% |
| 4B_1_S16 | Black | SRR14234415 | | 18.92 | 13.52 | 88.47% |
| 4B_2_S17 | Black | SRR14234414 | | 18.76 | 12.94 | 88.00% |
| 51R_1_S12 | Red | SRR14234419 | | 19.92 | 13.65 | 87.37% |
| 51R_2_S13 | Red | SRR14234418 | | 21.61 | 15.09 | 87.76% |
| 51R_3_S15 | Red | SRR14234416 | | 18.91 | 13.07 | 88.14% |
| 53R_2_S11 | Red | SRR14234420 | | 20.17 | 14.00 | 88.05% |
| 72R_1_S14 | Red | SRR14234417 | | 20.51 | 14.32 | 87.90% |

**Supplementary Table 2:** List of differentially expressed genes (DEGs) between red and black phenotypes reported with the log_2_Fold change(FC) across the treatments, FDR adjusted p-value, *B****.*** *impatiens* and *D. melanogaster* ortholog, and FlyBase ID. Genes colored in red are upregulated in the red form and those in black are upregulated in the black form. Genes are ordered by their adjusted p-value, with the most differentially expressed genes listed first.

| Rank | GeneID  (BIMP2.2) | log*_2_*  FC | padj | *B. impatiens* gene annotation | *D. melanogaster* ortholog | FlyBase ID |
| --- | --- | --- | --- | --- | --- | --- |
| 1 | LOC100749404 | 2.26 | 1.75E-52 | POU domain, class 2, transcription factor 2-like | nubbin (nub) | FBgn0085424 |
| 2 | LOC100740059 | 2.34 | 1.75E-42 | mycosubtilin synthase subunit C | ebony (e) | FBgn0000527 |
| 3 | LOC100742573 | 4.74 | 4.66E-35 | homeobox protein abdominal-B-like | abdominal B (Abd-B) | FBgn0000015 |
| 4 | LOC100750025 | -1.35 | 5.94E-29 | tyrosine 3-monooxygenase | pale (ple) | FBgn0005626 |
| 5 | LOC100740135 | -2.28 | 3.20E-24 | peroxidase | peroxidase (Pxd) | FBgn0004577 |
| 6 | LOC100742679 | 1.29 | 3.38E-21 | serine/threonine-protein kinase ICK | Mitogen-activated protein kinase (CG42366) | FBgn0259712 |
| 7 | LOC105680367 | 2.04 | 4.03E-21 | uncharacterized LOC105680367 | NA | NA |
| 8 | LOC100745478 | -0.56 | 6.28E-18 | ATP-citrate synthase | ATP citrate lyase (ATPCL) | FBgn0020236 |
| 9 | LOC100749393 | -1.45 | 1.19E-17 | myosin-IIIb | neither inactivation nor afterpotential C (ninaC) | FBgn0002938 |
| 10 | LOC100744015 | 2.29 | 1.22E-16 | uncharacterized LOC100744015 | CG13314 | FBgn0035949 |
| 11 | LOC100740591 | -3.02 | 2.16E-13 | uncharacterized LOC100740591 | NA | NA |
| 12 | LOC112213073 | 0.73 | 1.34E-12 | protein THEM6 | NA | NA |
| 14 | LOC100744811 | -1.34 | 2.88E-12 | uncharacterized LOC100744811 | CG31974 | FBgn0051974 |
| 13 | LOC100741205 | 1.06 | 2.88E-12 | fatty-acid amide hydrolase 2 | CG8839 | FBgn0033717 |
| 15 | LOC112213217 | -0.55 | 9.60E-12 | uncharacterized LOC112213217 | NA | NA |
| 16 | LOC105680269 | -1.02 | 9.62E-12 | uncharacterized LOC105680269 | NA | NA |
| 17 | LOC100742474 | 0.89 | 2.28E-11 | tetraspanin-9 | tetraspanin 5D (Tsp5D) | FBgn0029837 |
| 18 | LOC100748229 | 1.06 | 1.22E-10 | peroxidasin-like | Curly Su (cysu) | FBGN0038511 |
| 19 | LOC100744527 | 2.20 | 2.02E-10 | endocuticle structural glycoprotein SgAbd-1-like | Cuticular protein 67Fb (Cpr67Fb) | FBGN0036110 |
| 21 | LOC100742167 | 0.63 | 1.68E-09 | uncharacterized LOC100742167 | transcript 48 (T48) | FBgn0004359 |
| 20 | LOC100742962 | 1.76 | 1.68E-09 | uncharacterized LOC100742962 | NA | NA |
| 22 | LOC105681550 | -0.90 | 4.07E-09 | uncharacterized LOC105681550 | NA | NA |
| 23 | LOC100740670 | 1.10 | 5.72E-09 | 1,5-anhydro-D-fructose reductase | CG2767 | FBgn0037537 |
| 24 | LOC100748366 | -1.58 | 8.57E-09 | heterogeneous nuclear ribonucleoprotein A1 | NA | NA |
| 25 | LOC100742554 | 1.30 | 1.81E-08 | elongation of very long chain fatty acids protein AAEL008004-like | CG31522 | FBGN0051522 |
| 26 | LOC100746218 | 0.86 | 1.95E-08 | uncharacterized LOC100746218 | NA | NA |
| 27 | LOC105680989 | -0.75 | 7.69E-08 | nuclear RNA export factor 2-like | nuclear RNA export factor 2 (nxf2) | FBgn0036640 |
| 28 | LOC100741974 | 1.11 | 8.33E-08 | glycine-rich protein 5 | NA | NA |
| 29 | LOC100740006 | -1.71 | 1.19E-07 | uncharacterized LOC100740006 | NA | NA |
| 30 | LOC100743447 | -1.75 | 1.19E-07 | fatty-acid amide hydrolase 2-B | CG7910 | FBGN0037547 |
| 31 | LOC117151968 | -1.82 | 1.35E-07 | uncharacterized LOC117151968 | NA | NA |
| 32 | LOC100741285 | 1.22 | 1.35E-07 | phenoloxidase 1 | prophenoloxidase 3 (PPO3) | FBgn0261363 |
| 33 | LOC100742835 | 0.53 | 4.32E-07 | uncharacterized LOC100742835 | regeneration (rgn) | FBgn0261258 |
| 34 | LOC100740069 | -1.32 | 4.81E-07 | heparan sulfate 2-O-sulfotransferase pipe | pipe (pip) | FBgn0003089 |
| 35 | LOC100746202 | -0.62 | 1.01E-06 | sphingosine kinase 2 | Sphingosine kinase 2 | FBGN0052484 |
| 36 | LOC100740300 | -0.89 | 1.39E-06 | sodium-independent sulfate anion transporter-like | CG7912 | FBgn0039736 |
| 37 | LOC100746987 | -0.57 | 1.39E-06 | acyl-CoA Delta | CG9743 | FBgn0039756 |
| 38 | LOC100744559 | -0.44 | 1.39E-06 | zinc finger protein Noc | no ocelli (noc) | FBgn0005771 |
| 39 | LOC100747001 | -1.73 | 2.55E-06 | uncharacterized LOC100747001 | NA | NA |
| 40 | LOC100740183 | 0.80 | 2.85E-06 | DENN domain-containing protein 10 | NA | NA |
| 42 | LOC100742085 | -0.77 | 6.13E-06 | sodium-dependent nutrient amino acid transporter 1-like | Nutrient Amino Acid Transporter 1(NAAT1) | FBgn0029762 |
| 41 | LOC100743004 | -0.75 | 6.13E-06 | sodium-dependent nutrient amino acid transporter 1-like | CG1698 | FBgn0033443 |
| 43 | LOC100742789 | 0.47 | 8.47E-06 | protein THEM6 | CG4666 | FBgn0029838 |
| 44 | LOC100744689 | 2.00 | 1.73E-05 | uncharacterized LOC100744689 | CG15020 | FBgn0035543 |
| 45 | LOC100743603 | 0.34 | 2.34E-05 | breast cancer anti-estrogen resistance protein 1 | p130CAS | FBgn0035101 |
| 46 | LOC100744428 | -2.46 | 3.31E-05 | protein drumstick | drumstick (drm) | FBgn0024244 |
| 47 | LOC100741098 | 1.29 | 4.20E-05 | uncharacterized LOC100741098 | NA | NA |
| 48 | LOC105680786 | 2.99 | 4.86E-05 | pancreatic triacylglycerol lipase | CG17292 | FBGN0032029 |
| 49 | LOC100745887 | -0.45 | 6.15E-05 | E3 ubiquitin-protein ligase TRIM23 | NA | NA |
| 50 | LOC100743581 | -0.48 | 7.16E-05 | pyruvate kinase | pyruvate kinase (PyK) | FBgn0267385 |
| 51 | LOC100747239 | 0.65 | 8.41E-05 | protein apterous-like | apterous (ap) | FBgn0267978 |
| 52 | LOC112214031 | 2.96 | 8.73E-05 | fatty acyl-CoA reductase 1-like | NA | NA |
| 53 | LOC112212165 | -3.01 | 8.78E-05 | uncharacterized LOC112212165 | NA | NA |
| 54 | LOC100747372 | 0.29 | 9.76E-05 | disks large 1 tumor suppressor protein | discs large 1 (dlg1) | FBgn0001624 |
| 56 | LOC100744998 | 2.77 | 1.07E-04 | dynein-1-beta heavy chain, flagellar inner arm I1 complex | Dynein heavy chain at 62B | FBgn0013811 |
| 55 | LOC117151598 | 5.36 | 1.07E-04 | uncharacterized LOC117151598 | NA | NA |
| 57 | LOC100747133 | -0.42 | 1.20E-04 | bestrophin-2 | Bestrophin 1 | FBGN0040238 |
| 58 | LOC100743169 | 3.00 | 1.28E-04 | uncharacterized LOC100743169 | CG7730 | FBgn0036689 |
| 60 | LOC100747537 | -0.68 | 1.41E-04 | aminopeptidase N | CG40470 | FBgn0058470 |
| 59 | LOC100741805 | -0.34 | 1.41E-04 | organic cation transporter protein | CG6126 | FBgn0038407 |
| 61 | LOC112212541 | 1.77 | 1.41E-04 | uncharacterized LOC112212541 | NA | NA |
| 64 | LOC100742890 | -0.79 | 1.41E-04 | dual oxidase | dual oxidase (Duox) | FBgn0031464 |
| 63 | LOC100744646 | 2.81 | 1.41E-04 | endocuticle structural glycoprotein SgAbd-2-like | Cuticular protein 67Fb (Cpr67Fb) | FBGN0036110 |
| 62 | LOC112213049 | 3.31 | 1.41E-04 | odorant receptor Or2-like | NA | NA |
| 65 | LOC100745868 | -0.79 | 1.52E-04 | fibrillin-2-like | NA | NA |
| 66 | LOC100742291 | 0.88 | 1.57E-04 | MORN repeat-containing protein 3 | CG30429 | FBgn0050429 |
| 67 | LOC100748359 | -1.12 | 1.81E-04 | probable G-protein coupled receptor CG31760 | CG31760 | FBgn0051760 |
| 68 | LOC100747197 | -0.41 | 1.81E-04 | DNA-directed RNA polymerase III subunit RPC1 | RNA polymerase III subunit C160 (RpIIIC160) | FBgn0030687 |
| 69 | LOC100742783 | -0.35 | 2.33E-04 | multidrug resistance-associated protein 1 | Multidrug-Resistance like protein 1 (MRP) | FBgn0032456 |
| 70 | LOC100744087 | -0.28 | 2.33E-04 | valine--tRNA ligase | Valyl-tRNA synthetase (ValRS) | FBgn0027079 |
| 71 | LOC100744939 | 0.51 | 2.77E-04 | uncharacterized LOC100744939 | NA | NA |
| 72 | LOC100748750 | -0.41 | 2.92E-04 | hexosaminidase D | CG7985 | FBgn0028499 |
| 73 | LOC100744239 | -0.37 | 3.05E-04 | syntaxin-binding protein 5 | Tomosyn | FBgn0030412 |
| 74 | LOC100741712 | 1.57 | 3.32E-04 | protein lethal | lethal (2) essential for life | FBGN0011296 |
| 75 | LOC100746964 | -0.48 | 4.31E-04 | peroxisome biogenesis factor 10 | peroxin 10 (Pex10) | FBgn0035233 |
| 76 | LOC105681248 | -0.48 | 4.35E-04 | uncharacterized LOC105681248 | javelin-like (jvl) | FBgn0263929 |
| 77 | LOC100748853 | -0.30 | 4.68E-04 | tafazzin homolog | tafazzin (Taz) | FBgn0026619 |
| 78 | LOC100749847 | -0.35 | 5.03E-04 | protein toll | toll (Tl) | FBgn0262473 |
| 79 | LOC100740260 | 1.00 | 5.03E-04 | toll-like receptor Tollo | tollo (Tollo) | FBgn0029114 |
| 80 | LOC100742349 | -0.90 | 5.38E-04 | uncharacterized LOC100742349 | CG32225 | FBgn0052225 |
| 81 | LOC105682119 | -1.00 | 5.61E-04 | uncharacterized LOC105682119 | NA | NA |
| 82 | LOC100746168 | -0.77 | 6.27E-04 | sodium-dependent nutrient amino acid transporter 1-like | Nutrient Amino Acid Transporter 1 | FBGN0029762 |
| 83 | LOC100747875 | -0.55 | 6.37E-04 | inositol 1,4,5-trisphosphate receptor | Inositol 1,4,5,-trisphosphate receptor (Itp-r83A) | FBgn0010051 |
| 84 | LOC100749115 | -6.66 | 6.78E-04 | elongation of very long chain fatty acids protein 6-like | Baldspot | FBgn0260960 |
| 85 | LOC100744622 | -1.18 | 7.10E-04 | cytochrome P450 6k1-like | Cyp6g2 (Cyp6g2) | FBgn0033696 |
| 86 | LOC100745682 | 0.42 | 7.18E-04 | sushi | furrowed (fw) | FBgn0001083 |
| 88 | LOC105682151 | -0.52 | 8.13E-04 | cytochrome P450 9e2-like | Cyp9f2 (Cyp9f2) | FBgn0038037 |
| 87 | LOC100742268 | -0.37 | 8.13E-04 | Na | CG10939 | FBgn0010620 |
| 89 | LOC100743147 | 0.99 | 8.56E-04 | transient receptor potential-gamma protein | transient receptor potential cation channel gamma (Trpγ) | FBgn0032593 |
| 90 | LOC100748423 | -1.89 | 8.61E-04 | cadherin-23 | cadherin 88C (Cad88C) | FBgn0038247 |
| 92 | LOC117151447 | -1.17 | 8.67E-04 | uncharacterized LOC117151447 | NA | NA |
| 91 | LOC100749035 | 0.51 | 8.67E-04 | guanine deaminase | dihydropterin deaminase (DhpD) | FBgn0261436 |
| 93 | LOC117152388 | 3.26 | 8.82E-04 | fatty acyl-CoA reductase 1-like | NA | NA |
| 94 | LOC100742589 | 0.31 | 9.70E-04 | uncharacterized LOC100742589 | NA | NA |
| 95 | LOC100745112 | 0.64 | 9.70E-04 | ephrin type-B receptor 1-B | Eph receptor tyrosine kinase (Eph) | FBgn0025936 |

**Supplementary Table 3:** Functional annotation chart of enriched Gene Ontology (GO) terms obtained from DAVID v. 6.8 reported with their categories (i.e. functional annotation databases from which they were derived from) and associated p-values (EASE score; a modified fisher's exact test p-value where statistical significance level assessed at p < 0.05).

| Category | Term Description | Gene Count | P-Value |
| --- | --- | --- | --- |
| GOTERM_BP_DIRECT | Wing Disc Development | 6 | 7.29E-04 |
| GOTERM_BP_DIRECT | Dopamine Metabolic Process | 3 | 9.78E-04 |
| INTERPRO | Haem Peroxidase | 3 | 1.78E-03 |
| INTERPRO | Haem Peroxidase, Animal, Subgroup | 3 | 1.78E-03 |
| GOTERM_MF_DIRECT | Heme Binding | 5 | 1.81E-03 |
| UP_KEYWORDS | Membrane | 28 | 2.37E-03 |
| GOTERM_CC_DIRECT | Integral Component Of Membrane | 24 | 4.04E-03 |
| GOTERM_MF_DIRECT | Peroxidase Activity | 3 | 4.04E-03 |
| GOTERM_BP_DIRECT | Developmental Pigmentation | 3 | 5.17E-03 |
| UP_KEYWORDS | Monooxygenase | 4 | 5.33E-03 |
| UP_KEYWORDS | Transmembrane Helix | 25 | 5.33E-03 |
| UP_KEYWORDS | Transmembrane | 25 | 5.42E-03 |
| UP_KEYWORDS | Glycoprotein | 9 | 5.79E-03 |
| GOTERM_CC_DIRECT | Integral Component Of Plasma Membrane | 9 | 5.87E-03 |
| UP_KEYWORDS | Peroxidase | 3 | 6.38E-03 |
| UP_KEYWORDS | Lipid Biosynthesis | 4 | 8.03E-03 |
| UP_KEYWORDS | Alternative Splicing | 12 | 8.62E-03 |
| UP_KEYWORDS | Oxidoreductase | 9 | 9.36E-03 |
| UP_SEQ_FEATURE | Splice Variant | 12 | 1.02E-02 |
| UP_KEYWORDS | Fatty Acid Biosynthesis | 3 | 1.09E-02 |
| GOTERM_MF_DIRECT | Iron Ion Binding | 4 | 1.19E-02 |
| GOTERM_BP_DIRECT | Regulation Of Glucose Metabolic Process | 6 | 1.29E-02 |
| UP_SEQ_FEATURE | Glycosylation Site:N-Linked (Glcnac...) | 8 | 1.58E-02 |
| UP_KEYWORDS | Fatty Acid Metabolism | 3 | 1.64E-02 |
| GOTERM_BP_DIRECT | Embryonic Pattern Specification | 3 | 1.69E-02 |
| GOTERM_BP_DIRECT | Oxidation-Reduction Process | 7 | 1.79E-02 |
| UP_KEYWORDS | Lipid Metabolism | 4 | 2.40E-02 |
| UP_KEYWORDS | Developmental Protein | 10 | 2.48E-02 |
| GOTERM_BP_DIRECT | Adult Chitin-Containing Cuticle Pigmentation | 3 | 2.58E-02 |
| GOTERM_BP_DIRECT | Response To Oxidative Stress | 4 | 2.84E-02 |
| UP_SEQ_FEATURE | Topological Domain:Cytoplasmic | 7 | 2.97E-02 |
| GOTERM_BP_DIRECT | Long-Chain Fatty Acid Biosynthetic Process | 2 | 2.99E-02 |
| UP_KEYWORDS | Hydrogen Peroxide | 2 | 3.72E-02 |
| GOTERM_MF_DIRECT | Carbon-Nitrogen Ligase Activity, With Glutamine As Amido-N-Donor | 2 | 3.87E-02 |
| GOTERM_MF_DIRECT | Cation:Amino Acid Symporter Activity | 2 | 3.87E-02 |
| GOTERM_BP_DIRECT | Amino Acid Transmembrane Import | 2 | 3.97E-02 |
| UP_KEYWORDS | Receptor | 6 | 4.18E-02 |
| UP_SEQ_FEATURE | Site:Transition State Stabilizer | 2 | 4.37E-02 |
| INTERPRO | Amidase | 2 | 4.63E-02 |
| INTERPRO | Amidase Signature Domain | 2 | 4.63E-02 |
| UP_SEQ_FEATURE | Metal Ion-Binding Site:Iron (Heme Axial Ligand) | 3 | 4.77E-02 |
| GOTERM_MF_DIRECT | Amidase Activity | 2 | 4.81E-02 |
| UP_KEYWORDS | Secreted | 4 | 4.84E-02 |

**Supplementary Table 4:** Welch’s two-sample t-test results (p-values) conducted on stage-specific black and red morph log2 fold difference data for each gene and caste (male/worker). Two-sample t-tests were used for 0hr worker stages as there is only one replicate for black morphs.

| Gene | Caste | Stage | P-value |
| --- | --- | --- | --- |
| *nubbin* | male | P13 | 0.6494 |
| *nubbin* | male | P15 | 0.8781 |
| *nubbin* | male | 0hr | 0.001137 |
| *nubbin* | male | 6hr | 0.003728 |
| *nubbin* | male | 12hr | 0.04565 |
| *nubbin* | male | 24hr | 0.1029 |
| *ebony* | male | P13 | 0.7132 |
| *ebony* | male | P15 | 0.8108 |
| *ebony* | male | 0hr | 0.000961 |
| *ebony* | male | 6hr | 0.002095 |
| *ebony* | male | 12hr | 0.002118 |
| *ebony* | male | 24hr | 0.2375 |
| *pale* | male | P13 | 0.484 |
| *pale* | male | P15 | 0.6706 |
| *pale* | male | 0hr | 0.5514 |
| *pale* | male | 6hr | 0.6581 |
| *pale* | male | 12hr | 0.8761 |
| *pale* | male | 24hr | 0.2867 |
| *Abd-B* | male | P13 | 0.2148 |
| *Abd-B* | male | P15 | 0.03038 |
| *Abd-B* | male | 0hr | 0.000113 |
| *Abd-B* | male | 6hr | 0.0116 |
| *Abd-B* | male | 12hr | 0.01316 |
| *Abd-B* | male | 24hr | 0.1059 |
| *ebony* | worker | P15 | 0.9932 |
| *ebony* | worker | 0hr | 0.009175 |
| *nubbin* | worker | P15 | 0.2055 |
| *nubbin* | worker | 0hr | 0.06033 |
| *pale* | worker | P15 | 0.9983 |
| *pale* | worker | 0hr | 0.1069 |

**Supplementary Table 5:** A table reporting information about the samples in q-PCR assay. Corresponding Sample IDs, developmental stage, and phenotypes were reported form each sample.

| Sample # | Sample ID | Phenotype | Stage |
| --- | --- | --- | --- |
| 1 | 38-S13-male1 | Black | p13 |
| 2 | 58-S13-male-1 | Black | p13 |
| 3 | 58-S13-male-3 | Black | p13 |
| 4 | 38-S13-male-2 | Black | p13 |
| 5 | 155-S13-male-2 | Red | p13 |
| 6 | 131-S13-male-1 | Red | p13 |
| 7 | 133-S13-male-2 | Red | p13 |
| 8 | 133-S13-male-1 | Red | p13 |
| 9 | 38-S15-male-1 | Black | p15 |
| 10 | 58-s15-male-2 | Black | p15 |
| 11 | 38-s15-male-2 | Black | p15 |
| 12 | 136-S15-male-2 | Black | p15 |
| 13 | 134-s15-male-1 | Red | p15 |
| 14 | 155-s15-male-3 | Red | p15 |
| 15 | 155-S15-male-2-2 | Red | p15 |
| 16 | 119-S15-male-1 | Red | p15 |
| 17 | 136-0hcallow-male-2 | Black | Callow |
| 19 | 38-0hcallow-male-3 | Black | Callow |
| 20 | 58-0hcallow-male-2 | Black | Callow |
| 21 | 155-0hcallow-male-2 | Red | Callow |
| 22 | 123-0hcallow-male-1&2 | Red | Callow |
| 23 | 155-0hcallow-male | Red | Callow |
| 24 | 109-0hcallow-male | Red | Callow |
| 25 | 42-6hour callow hc | Black | 6hr |
| 26 | 42-6hour callow hc | Black | 6hr |
| 27 | 133-6hc-male-4 | Red | 6hr |
| 28 | 133-6hc-male-4 | Red | 6hr |
| 29 | 133-6hc-male-1 | Red | 6hr |
| 30 | 42-12hc-male-4 | Black | 12hr |
| 31 | 42-12hc-male-5&2 | Black | 12hr |
| 32 | 133-12hc-male-1&2 | Red | 12hr |
| 33 | 133-12hc-male-3 | Red | 12hr |
| 34 | 42-24hc-male-2&4 | Black | 24hr |
| 35 | 42-24hc-male-3&2 | Black | 24hr |
| 36 | 133-24hc-male1 | Red | 24hr |
| 37 | 133-24hc-male-3 | Red | 24hr |
| 38 | 133-24hc-male-2 | Red | 24hr |

**Supplementary Figure 1:** A hierarchical clustering heatmap for the differentially expressed genes (n=95) using normalized count data. Each column represents transcriptome samples and each row individual genes. The color scale represents Z-score value range, with green depicting downregulation and red upregulation. Two clusters shaded grey on the gene annotation depict correlation among the most upregulated melanin pathway candidate genes (i.e. *ebony* and *pale*) in red and black forms respectively.

**
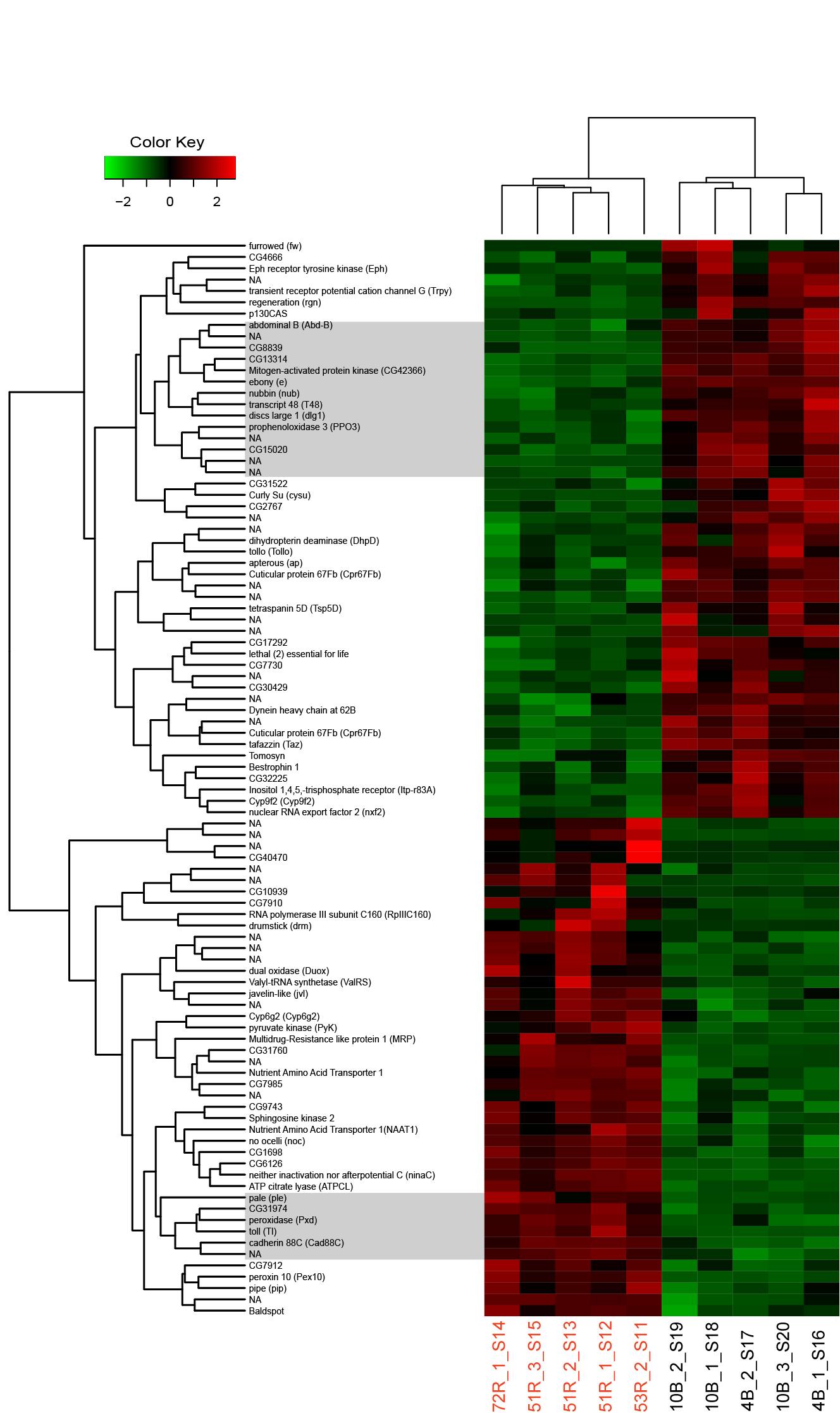
**
