# Supplementary material for: Developmental Transcriptomics Reveals a Gene Network Driving Mimetic Color Variation in a Bumble Bee
Source: Genome Biol Evol. 2021 Apr 21;13(6):evab080. doi: 10.1093/gbe/evab080 (PMC8220310; doi:10.1093/gbe/evab080)
Supplement: evab080_supplementary_Data [file evab080_supplementary_data.zip › graphicabstract_transcriptomemel.pdf]

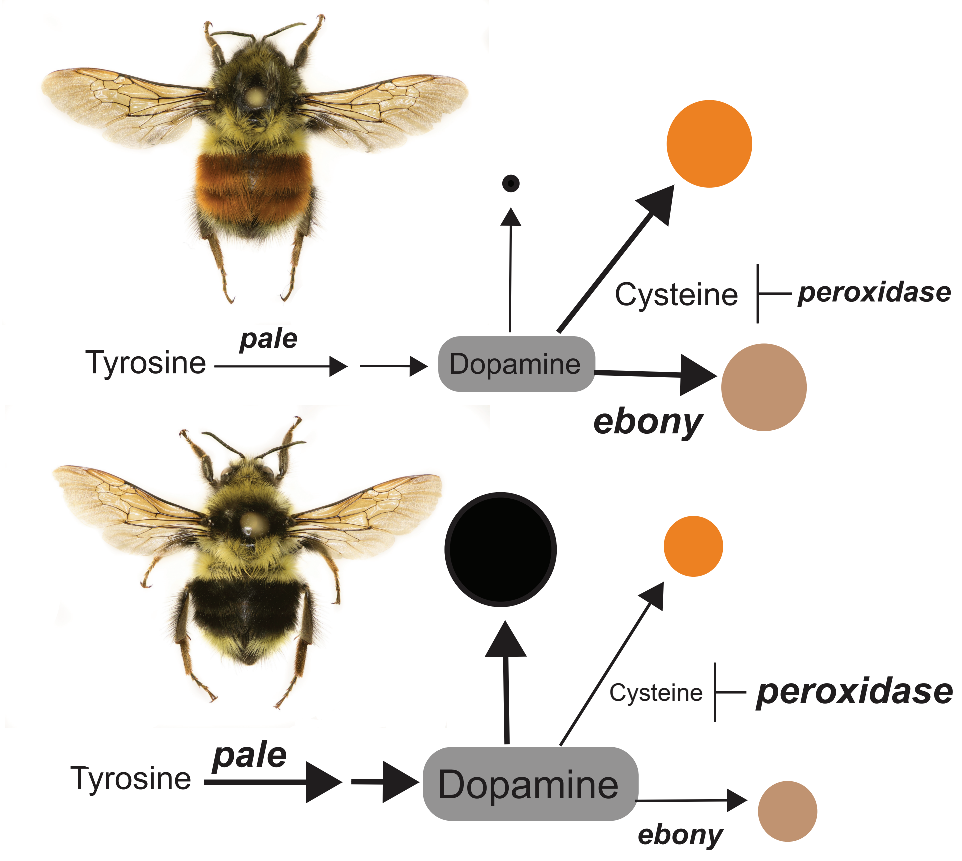

pale
Tyrosine
Dopamine
Cysteine
peroxidase
ebony
pale
Tyrosine
Dopamine
Cysteine
peroxidase
ebony
